# Antioxidant Effect of MnTE-2-PyP on Lung in Asthma Mice Model

**DOI:** 10.1100/2012/379360

**Published:** 2012-04-30

**Authors:** Lyudmil Terziev, Violeta Dancheva, Veneta Shopova, Galya Stavreva

**Affiliations:** ^1^Sector of Clinical Immunology and Allergology, Pelven Medical University, 5800 Pleven, Bulgaria; ^2^Sector of Disaster Medicine, Pelven Medical University, 5800 Pleven, Bulgaria; ^3^Sector of Clinical and Experimental Pharmacology, Pelven Medical University, 5800 Pleven, Bulgaria

## Abstract

*Aim*. To investigate the effects of MnTE-2-PyP on some markers of antioxidant defence system in asthma mice model. *Material and Methods*. The animals were divided into four groups: group 1, controls; group 2, injected with ovalbumin, group 3, treated with MnTE-2-PyP, and group 4, treated with ovalbumin and MnTE-2-PyP. The activities of superoxide dismutase, catalase, glutathione peroxidase and nonprotein sulfhydryl groups content (NPSH) were determined in lung homogenate. *Results*. The activities of superoxide dismutase and catalase in group 2 decreased significantly as compared to control group. The decrease of the same enzymes in group 4 was lower and significant as compared to group 2. Changes in the glutathione peroxidase activity showed a similar dynamics. The NPSH groups content decreased in group 2. In group 4 this decrease was relatively lower as compared to group 2. *Conclusions*. The application of MnTE-2-PyP mitigated the effects of oxidative stress in asthma mice model.

## 1. Introduction

Asthma is a major, worldwide health concern, affecting children and adults. It affects 7% of the US population and 300 million worldwide [1]. Currently in the USA, some 22 million people are reported to have asthma, six million of whom are children [2]. It is among the commonest chronic conditions in Western countries affecting 1 in 7 children and 1 in 12 adults (equivalent to 5.1 million people in the UK) and is responsible each year for 1500 avoidable deaths, as well as 20 million lost working days. The annual UK healthcare cost is estimated to be *£*2.5 billion [3]. Oxidative stress is believed to play a role in the development of number of human diseases such as cardiovascular disorders, immunologic diseases, cancer, and asthma. A large amount of epidemiological and clinical evidence exists to support the relationship between increased reactive oxygen species (ROS) and the pathogenesis of bronchial asthma [4–8]. Oxidative stress is a deleterious process that leads to lung damage and consequently to various lung diseases. To protect against exposure to oxidants the lungs have a powerful antioxidant system, including nonenzymatic and enzymatic antioxidants, which may delay or prevent oxidation, but also eliminate reactive oxygen species [5]. At high levels of oxidative stress, however, antioxidants become depleted, and an imbalance between oxidants and antioxidants occurs, which causes pathological damage, or a variety of cellular responses through formation of secondary ROS [9]. All the major varieties of inflammatory lung diseases, asthma, chronic obstructive pulmonary disease, idiopathic pulmonary fibrosis, acute respiratory distress syndrome, interstitial lung diseases, and bronchopulmonary dysplasia share a common feature of impaired oxidant/antioxidant ratio [10]. Therefore, the supplementation of antioxidants to boost the endogenous antioxidants or scavenge excessive ROS production could be utilized to prevent the inflammatory response in asthma by restoring oxidant-antioxidant balance. Current knowledge of the effects of oxidative stress allow the development of new classes of antioxidants in the treatment of asthma and other disorders, associated with the oxidative stress. Considerable progress has been made in the last years, in developing mitochondria-targeted antioxidants such as manganese porphyrins [2]. A number of water-soluble *meso*-substituted manganese porphyrins with a molecular weight above 800 quickly pass through the cell membranes and are distributed into the mitochondria [11, 12]. Therefore we aimed to study the effect of MnTE-2-PyP (Manganese (III) 5,10,15,20-tetrakis (N-ethylpyridinium-2-yl) porphyrin), a manganese-*meso-*porphyrin also known as AEOL-10113, on some markers of lung antioxidant defence system in asthma mice model.

## 2. Material and Methods

### 2.1. Chemicals

Ovalbumin, grade V and phosphate-buffered saline (PBS) were purchased from the Sigma-Aldrich Company and Imject Alum was obtained from Pierce Chemical Company (USA).

MnTE-2-PyP was kindly provided by Ines Batinić-Haberle from the Department of Radiation Oncology, Duke University Medical Center, Durham, NC, USA.

### 2.2. Animals and Experimental Protocol

The experiment was performed in accordance with the Animal Welfare Regulations and was approved by the University Ethics Committee.

The study was carried out on 24 female C57Bl/6 mice (weight 20 ± 2.0 g, 8–10 weeks old). The animals were raised at the university vivarium at a temperature of 22 ± 2°C and humidity of 50 ± 10%, given normal pelleted diet and water *ad libitum*. The mice were divided into four groups: group 1, controls; group 2, injected with ovalbumin (OVA); group 3, treated with MnTE-2-PyP; group 4, treated with OVA and MnTE-2-PyP. The animals from groups 1 and 3 were injected *i.p*. with a 100 *μ*L phosphate-buffed saline (PBS) + Imject Alum (1 : 1) on days 0 and 14. The animals from groups 2 and 4 were injected with a 100 *μ*L ovalbumin solution, containing 20 *μ*g OVA on the same days. On days 24, 25, and 26, mice from groups 1 and 3 were given inhalation with PBS for 30 min, and those from groups 2 and 4 were given inhalation with a 1% ovalbumin solution (OVA dissolved in PBS). For this purpose, a special plexiglass chamber was used. One hour before inhalation, and 12 hours later the animals from groups 1 and 2 were injected *i.p*. with 100 *μ*L PBS, and those from groups 3 and 4 received a 100 *μ*L MnTE-2-Pyp dissolved in PBS, containing 5 mg/kg, that is, the total daily dose was 10 mg/kg. The experimental protocol was represented in Table 1.

The solution was sterilized by filtration through 0.2 *μ*m filters.

### 2.3. Biochemical Assays in the Lung Homogenate

The animals were sacrificed on day 28 (48 hours after the last inhalation) under thiopental anesthesia (50 mg/kg). The chest was opened and the lungs were perfused *in situ* via the right heart ventricle with saline (10 mL). The right lung was ligated at the hilus, cut, and then removed from the chest and used to prepare the lung homogenate. The tissue was homogenized with ice-cold 0.25 M sucrose in Tris HCl, pH = 7.4, in 1 : 10 ratio. The homogenate was centrifuged (9000 ×g, 30 min), and the supernatant was stored on ice. The superoxide dismutase (SOD) activity in U/mg lung tissue was determined by the method of Maral et al. [13], and catalase (CAT) activity in mcat/g tissue was assessed by the method of Koroljuk et al. [14]. The activity of glutathione peroxidase (GP) in U/g lung tissue was measured by the method of Bernchnaider, modified by Pereslegina [15]. The non-protein sulfhydryl (NPSH) groups content in mol × 10^7^/g tissue was measured by the method of DeLucia et al. [16].

### 2.4. Statistical Analysis

Experimental data were analyzed using SPSS 14. When we tested for normality, two variables-GP and CAT showed non-parametric distribution, and we used medians, interquartile range and Mann-Whitney test for comparison. For the rest of the variable we applied post-hoc ANOVA test and data were presented as mean ± standard error of mean (SEM). *P* < 0.05 were considered statistical significant.

## 3. Results

**Figure 1:**
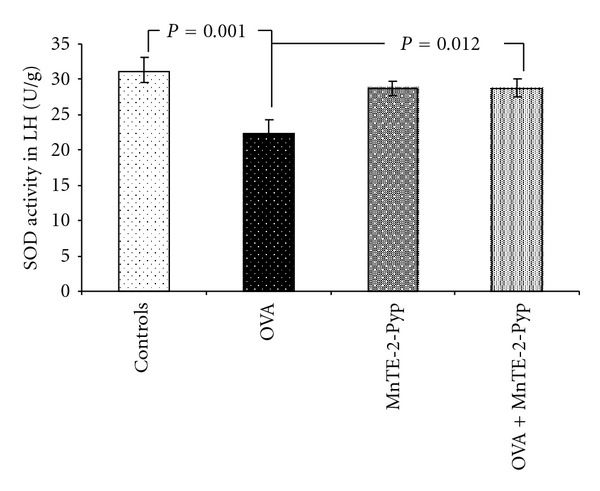
Activity of superoxide dismutase in lung homogenate. Each point represents the mean ± SEM for six mice.

**Figure 2:**
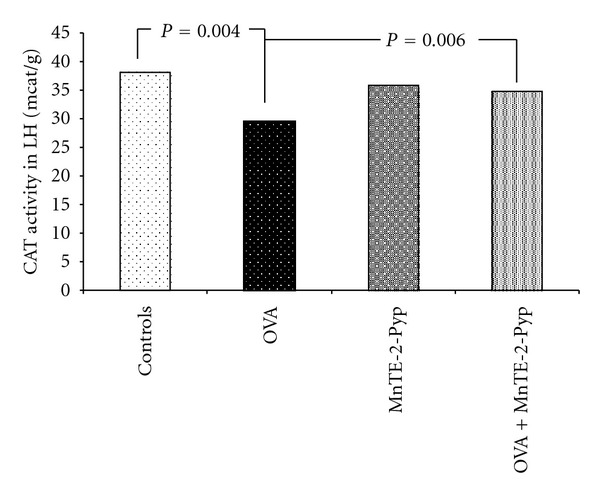
Activity of catalase in lung homogenate. Each point represents the median for six mice.

**Figure 3:**
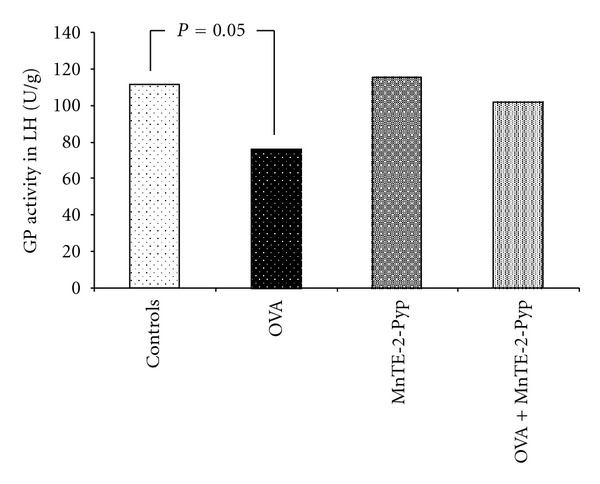
Activity of glutathione peroxidase in lung homogenate. Each point represents the median for six mice.

**Figure 4:**
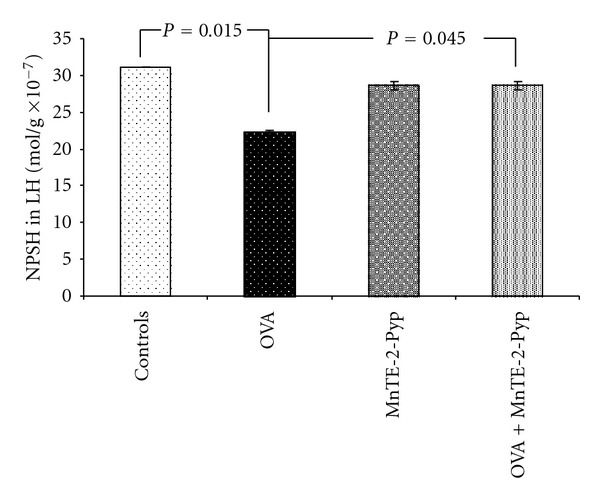
Content of nonprotein sulfhydryl groups in lung homogenate. Each point represents the mean ± SEM for six mice.

The activities of superoxide dismutase and catalase in group 2 (asthma-induced) decreased significantly in the lung homogenate up to 71% (*P* = 0.001) and up to 77% (*P* = 0.004) respectively, as compared with the controls. The decrease of the same parameters in group 4 was lower (92%, *P* = 0.012) than that in group 2 and 91% (*P* = 0.006), statistically significant compared to the group 2, respectively (Table 2, Figures 1 and 2). Changes in the glutathione peroxidase activity showed a similar dynamics, such as a decrease in the OVA group (68%) and in the values approximate to those of the controls in group 4. The decrease was 23% less than that in group 2 but it was non significant. (Table 2, Figure 3). The non-protein sulfhydryl (NPSH) groups content in the lung homogenate decreased up to 68% in group 2 (*P* = 0.015), and in the group treated with OVA and antioxidant (group 4) this decrease was relatively lower (*P* = 0.045) as compared to the OVA group (Table 2, Figure 4). The changes of the parameters in group 3 (MnTE-2-PyP alone) did not show significant changes compared to controls (Figures 1–4). 

## 4. Discussion

The decrease of the activity of key antioxidant enzymes in the lung such as SOD, CAT and GP, as well as the level of the non-protein sulfhydryl groups in animals injected and inhaled with OVA supports allegations that ovalbumin can provoke asthma, an oxidative stress-associated disease [3, 4, 6, 17]. Some studies had revealed suppressed activity of catalase, superoxide dismutase and glutathione peroxidase in patients with bronchial asthma [18]. Comhair et al. also showed that the antioxidant enzymes (SOD and CAT) are in lower levels in asthmatic patients [19, 20]. The activity of GPx has been found to be much lower in asthmatic children compared to normal children [21].

MnTE-2-PyP has a beneficial effect on the activity of studied antioxidant enzymes. Metalloporphyrins, and preferable water-soluble Mn complexes, remain the most stable and the most active prospective SOD mimetics [22]. That is way we chose and studied the effects of this compound. The activity of some manganese porphyrins approaches that of the SOD enzymes themselves [23]. Over the year, views of the researchers evolved from SOD mimics, to  O_2_^−^/ONOO^−^ scavengers, and finally to redox modulators of cellular transcriptional activity [22]. Therefore they have at least four antioxidant properties, such as the removal of superoxide (O_2_^−•^), hydrogen peroxide (H_2_O_2_), peroxinitrite (ONOO^−^), and lipid peroxides [24, 25]. Given *i.p*. to mice at 10 mg/kg MnTE-2-PyP^5+^ distributed into all organs studied (liver, kidney, spleen, lung, heart, and brain). It had high chances to enter mitochondria. At the upper dose MnTE-2-PyP injection was found in mitochondria at 2.9 ng/mg protein [26]. Such levels are high enough to protect mitochondria against peroxynitrite-mediated damage [27]. The plasma half-life is about 1 h, and the organ half-life is about 60–135 h [22]. This explains why we have applied the antioxidant twice daily at intervals of 12 hours. The lack of decrease in activity of SOD in the group of animals, treated with OVA + MnTE-2-PyP is explained by the fact that *in vivo *they will be readily reduced by cellular reductants, flavoenzymes, NO, and so forth, to Mn(II)P [27, 28], which will then in turn reduce  O_2_^−•^  to H_2_O_2_, acting as superoxide reductases rather than SOD [29]. Moreover all synthetic SOD mimics can scavenge peroxynitrite or its degradation products [22]. MnTE-2-PyP is 16-fold more prone to oxidative degradation than other Mn porphyrins, the commercial, MnTBAP [30]. The effects, observed after Mn porphyrins use, were the consequence not only of mere scavenging of ROS/RNS (reactive nitrogen species), but also of Mn porphyrins being able to modulate ROS/RNS-based signaling pathways. Mn(III) *N*-alkylpyridylporphyrins inhibit *in vitro* and *in vivo* activation of several redox-controlled transcription factors such as HIF-1*α*  (hypoxia inducible factor 1*α*), NF-kB (nuclear factor kappa-B), AP-1 (activating protein-1), and SP-1 (specificity protein-1) [31, 32]. MnTE-2-PyP, a potent SOD mimic/ONOO^−^ scavenger, can strongly inhibit excessive activation of redox-sensitive cellular transcriptional activity, particularly suppressing hypoxia inducible factor 1*α* (HIF-1*α*) activation [33]. This results in lowering the number of inflammatory cells and cytokines, which in turn could lower to the levels of secondary ROS/RNS [34]. Manganese *meso*-porphyrins have been used successfully to treat oxidative stress in the numerous disorders such as stroke [35], spinal cord injury [36], Parkinson disease [37], Alzheimer disease [38], diabetes [39], cancer [40, 41], ischemia/reperfusion conditions [42, 43], bronchopulmonary dysplasia [44], asthma [45, 46], lung fibrosis [47], lung radioprotection [48], and sepsis [49]. The recent study on the effects of antioxidants, especially mitochondria-targeted antioxidants, indicated that these compounds may potentially improve the treatment of widespread and socially significant diseases, such as asthma.

## 5. Conclusion

The intraperitoneal application of 5 mg MnTE-2-PyP, a manganese *meso-*porphyrin, twice daily one hour before nebulization with ovalbumin on days 24, 25, and 26 mitigated the effects of the oxidative stress in asthma mice model assessed by key antioxidant enzymes and content of nonprotein sulfhydryl groups in the lungs. MnTE-2-PyP restored the basic antioxidant enzymes, such as superoxide dismutase, glutathione peroxidase as well as the content of the nonprotein sulfhydryl groups in the lungs.

## Figures and Tables

**Table 1:** Experimental protocol.

Groups	Treatment		
	Allergen	Sensitization/exposure (on days 0 and 14)	Exposure/challenge (on days 24, 25, and 26)

Group 1		Saline (i.p.)	Saline inhalation for 30′. Saline (i.p.) 1 hour before nebulization and 12 hours later)

Group 2	OVA	OVA/alum (i.p.)	OVA inhalation for 30′ Saline (i.p.) 1 hour before nebulization and 12 hours later)

Group 3		Saline (i.p.)	Saline inhalation for 30′. MnTE-2-PyP (i.p.) 1 hour before nebulization and 12 hours later)

Group 4	OVA	OVA/alum (i.p.)	OVA inhalation for 30′. MnTE-2-PyP (i.p.) 1 hour before nebulization and 12 hours later

**Table 2:** Effect of MnTE-2-PyP on the activity of some enzymes of lung antioxidant defence system in mice asthma model.

28 day after treatment (48 hours after the last inhalation)				
Parameters	Groups			
	Control	OVA	MnTE-2-PyP	OVA + MnTE-2-PyP

SOD activity in U/g	31.1 ± 1.79	22.1 ± 1.94*	28.6 ± 1.01	28.6 ± 1.25^†^
mean ± SEM				

CAT activity in mcat/g	38.14	29.48*	35.91	34.81^†^
Median (min–max)	(33.08–44.12)	(21.51–31.20)	31.92–38.72	31.69–43.51
Q_3_–Q_1_	5.07	4.30	4.5	9.14

GP activity in U/g	111.2	76.07*	115.3	101.5
Median (min–max)	51.4–138.74	66.85–83.56	100.7–118.25	62.27–136.92
Q_3_–Q_1_	29.65	6.38	12.74	49.81

NPSH groups in mol/g 10^−7^	0.58 ± 0.03	0.40 ± 0.03*	0.54 ± 0.084	0.55 ± 0.04^†^
mean ± SEM				

OVA: ovalbumin; MnTE-2-PyP: Manganese (III) 5,10,15,20-tetrakis (N-ethylpyridinium-2-yl) porphyrin; SOD: superoxide dismutase; CAT: catalase; GP: glutathion peroxidase; NPSH groups: non protein sulfhydryl groups; SEM, standard error of mean; Q_3_–Q_1_: interquartile range

*Different from control at *P* < 0.05;

^†^Different from group 2 (OVA) at *P* < 0.05.
